# Cardiac Rehabilitation to Inpatient Heart Transplant—HRN4HTx Intervention Protocol

**DOI:** 10.3390/nursrep14030155

**Published:** 2024-08-23

**Authors:** Maria Loureiro, Vítor Parola, João Duarte, Isabel Oliveira, Margarida Antunes, Gonçalo Coutinho, Maria Manuela Martins, André Novo

**Affiliations:** 1 Instituto Ciências Biomédicas Abel Salazar, Cintesis-NursID, Unidade Local de Saúde de Coimbra, 3000-602 Coimbra, Portugal; 2 Nursing School of Coimbra (ESEnfC), The Health Sciences Research Unit-Nursing (UICISA:E), Centre for Evidence-Based Practice—A Joanna Briggs Institute Centre of Excellence, 3004-011 Coimbra, Portugal; vitorparola@esenfc.pt; 3 Unidade Local de Saúde de Coimbra, 3000-602 Coimbra, Portugal; 7220@ulscoimbra.min-saude.pt; 4 Nursing School of Coimbra (ESEnfC), The Health Sciences Research Unit-Nursing (UICISA:E), 3004-011 Coimbra, Portugal; ijoliveira12@gmail.com; 5 Unidade Local de Saúde de Coimbra, Núcleo de Investigação em Enfermagem do CHUC, 3000-602 Coimbra, Portugal; 6392@ulscoimbra.min-saude.pt; 6 Faculdade de Medicina de Coimbra, Unidade Local de Saúde de Coimbra, 3000-602 Coimbra, Portugal; 8058@ulscoimbra.min-saude.pt; 7 Escola Superior de Enfermagem do Porto, Cintesis-NursID, 4200-450 Porto, Portugal; mmmartins1956@gmail.com; 8 Instituto Politécnico de Bragança-Escola Superior de Saúde, LiveWell—Research Center for Active Living and Wellbeing, 5300-121 Bragança, Portugal; andre@ipb.pt

**Keywords:** cardiac rehabilitation, heart transplantation, inpatient, rehabilitation nursing

## Abstract

Heart transplantation is the gold-standard treatment for terminal heart failure. Despite being successful, pre- and post-transplant limitations interfere with patients’ functional capacity, self-care, and quality of life post-surgery. Rehabilitation is necessary to address these limitations, prevent complications, and promote a safe return home. This study analyzes the safety of a phase 1 cardiac rehabilitation protocol (RN4HTx) in heart transplant patients and its effects on self-care capacity. A quantitative, descriptive study was conducted with 19 heart transplant recipients. The protocol was implemented in collaboration with a rehabilitation professional, who monitored adverse events, hemodynamic variables, self-care capacity (Barthel) pre- and post-transplant, and functional capacity at discharge (6 min walk test). The results showed that 68.42% of recipients were men, with an average age of 50.21 years and 15 days of hospitalization post-transplant. Approximately 73.68% of recipients were transferred from other wards with changes in functional capacity. All patients progressed to the final stage of the program without adverse events. There was a notable improvement in self-care capacity before and after transplantation, with a measure of functional status of 310.035 m (6MWT). The study found that RN4HTx is a feasible cardiac rehabilitation program without adverse events in the immediate postoperative period following heart transplantation, positively impacting functional recovery and therapeutic self-care capacity, thus increasing the safety of returning home. This study was retrospectively registered on Clinical Trials—NCT06552390.

## 1. Introduction

Heart transplantation is considered an efficient gold-standard treatment for people with terminal heart failure (HF) [1]. The majority of pre-transplant patients present intolerance to activity that limits their functional capacity and independence in self-care [2], with functional status representing a strong predictor of post-transplant survival [3]. In addition to this functional disability, many of these patients are hospitalized to treat signs or symptoms of HF (with therapeutic support and/or assistive devices) [1] which cause mobility restrictions. These clinical changes resulting from HF induce functional limitations marked by negative implications for self-care, quality of life, and increased risk of mortality [3].

These preconditions are worsened by the effects of surgery, prolonged hospitalization, and complex medication regimens (such as immunosuppression). In the post-transplantation phase, there may be an increase in complications associated with immobility (e.g., myopathy), together with metabolic disorders, nutritional deficits, water restriction, and sometimes concomitant mood disorders, contributing to activity limitations that can impair function and quality of life, often for years after transplantation [4].

It should also be noted that heart transplantation continues to be a complex surgery with possible short- and long-term complications, such as early graft dysfunction, acute allograft rejection, and cardiac allograft vasculopathy, as well as non-graft-related complications such as infections, acute and chronic kidney injuries, and tumors [5]. All of these complications normally imply greater morbidity and mortality [6], tending to significantly reduce the potential for recovery and functionality [7] of the transplanted person.

In addition to the pathophysiological processes described above, it should be taken into account that after heart transplantation, cardiac denervation makes cardiorespiratory control (maximum O_2_-VO_2max_ consumption) and hemodynamic control (heart rate, cardiac output, and blood pressure) initially dependent on the Frank–Starling mechanism (law of preload dependent on venous return), circulating catecholamines (influence inotropism), and afterload (through the previous factors, they cause variations in the ejection fraction) due to the absence of sympathetic and parasympathetic stimulation and the baroreflex. Thus, people with heart transplants have lower VO_2max_ values (around 70–80% of the value predicted for their age concerning healthy people) and high values of heart rate, blood pressure, and vascular resistance at rest; during exercise, there is a weak increase in heart rate and blood pressure, associated with an increase in vascular resistance [8,9,10].

Cardiac rehabilitation is a multifactorial intervention process through which the individual maintains or recovers their physical, psychological, social, and work conditions satisfactorily after a cardiac event or in the context of chronic heart disease. This is based on the practice of adapting physical exercise and changing behaviors [11], oriented to trigger beneficial lifestyle changes, reduce and control risk factors, and intervene on psychological factors to reverse or delay the progression of underlying cardiovascular disease. Cardiac rehabilitation (CR) for people undergoing cardiac surgery is a safe and beneficial component of postoperative care [12,13,14], preventing complications and improving the person’s functional status with a positive impact on their quality of life. The main objectives of CR for people undergoing conventional heart surgery, which can be extrapolated to the particularity of the transplanted person, are improving functional capacity; early mobilization; achieving independence in self-care; preventing physical and psychological complications [15] resulting from post-cardiac surgery and immunosuppressive medication; [16] controlling cardiovascular risk factors; and promoting an early return to the community with referral to phase 2 programs.

Cardiac rehabilitation, with the integration of an exercise component, has a positive impact on people with heart transplants [1], as it improves maximum oxygen consumption, maximum heart rate, activity tolerance, autonomic function, functional independence, and quality of life [1,17]. As such, CR programs offer a promising modality for improving outcomes during the months after transplantation, particularly if they are started early.

There is growing interest in the role of cardiac rehabilitation in the postoperative period of heart transplantation, but the available literature remains limited. Most research to date has focused on phase 2 of CR, the moment after hospital discharge when patients are already stabilized. In this sense, an attempt was made to analyze the safety of a phase 1 cardiac rehabilitation protocol in people with heart transplants and its effects on self-care capacity.

## 2. Materials and Methods

A descriptive study was carried out, which took place between January 2021 and August 2023 at the reference center for heart transplantation at the Centro Hospitalar e Universitário de Coimbra, which is integrated into the cardiothoracic surgery unit. The study was approved by the hospital ethics committee, and all participants provided written consent before participating. The trial was retrospectively registered in Clinical Trials with the identifier NCT06552390.

### 2.1. Participants

All transplant patients were evaluated by a physician, a cardiac surgeon, and a rehabilitation nurse to determine whether they met the inclusion criteria. The inclusion criteria were (a) heart transplant patients; (b) over 18 years of age; and (c) the ability to provide informed consent. The exclusion criteria were (a) rejection—2R; (b) inability to understand the teachings/information and/or exercises due to cognitive impairment; (c) patients with external ventricular assistance; and (d) patients with an infectious process with hemodynamic changes, with 15 patients being excluded from a total of 34 transplants in this period. Sociodemographic variables (age, gender), number of days of hospitalization, and origin of admission (admission, home) were assessed for all participants, with the Barthel Index being measured on the date of admission and discharge. At the last moment, a 6 min gait test was also carried out.

The Barthel Index is an instrument that assesses a person’s level of independence when performing 10 ADLs: eating, hygiene care, using the toilet, bathing, dressing and undressing, sphincter control, walking, transferring from chair to chair/bed, and climbing stairs. The minimum score of 0 corresponds to maximum dependence for all ADLs assessed, and the maximum score of 100 is equal to total independence. It is an instrument that is easy to apply and interpret. Quick completion, low application cost, and the possibility of periodic repetition (which allows longitudinal assessment) without risk are some characteristics that make this scale one of the most used in clinical trials to assess the degree of dependence in performing 10 basic ADLs, which makes it the desirable choice in the post-heart transplant population.

The 6 min walk test can be used as a tool to measure a patient’s functional status, especially in the case of advanced diseases with multiple comorbidities that make them unable to perform more complex exercise tests. Although the 6 min walk test (6 MWT) provides information on functional capacity, response to therapy, and prognosis in a range of clinical conditions, the primary outcome is used to prescribe the intensity of physical training and as an outcome measure for cardiac rehabilitation [18,19].

### 2.2. Intervention Protocol

The intervention protocol was conducted during the hospitalization period at the reference center, integrated into the cardiothoracic surgery unit with interdisciplinary coordination of the transplant team, and designated Hospital Rehabilitation Nursing for Heart Transplant (HRN4HTx). HRN4HTx is a phase 1 rehabilitation intervention protocol for post-heart transplant (in the specific transplant unit), focused on the physical exercise component and an educational plan regarding risk factors and safety criteria. The intervention protocol and its assessments were implemented by the team’s rehabilitation nurses, always safeguarding the protective isolation required for these patients. The physical training protocol was divided into five stages of progressive levels of intervention (Table 1).

The protocol was defined following FITT parameters, international recommendations, and an integrated teaching program aimed at the therapeutic regimen exercise area. The frequency of intervention sessions was 7 days a week, twice a day (average of 13 sessions per week) throughout the hospitalization, with the type of interventions and their duration described in Table 1. The intensity was defined according to Borg’s modified scale of subjective perception of exertion (SPE), which must be maintained at a level less than 5. The talk test is low intensity; attention was paid to surgical edging through sternotomy due to the risk of dehiscence [15]. Heart rate was not considered an intensity definition parameter considering that chronotropic incompetence post-transplantation is expected, being analyzed as a safety/adverse event criterion.

Hemodynamic parameters and the subjective notion of effort were measured during rehabilitation sessions to measure the presence of adverse events. Adverse events (AEs) and serious adverse events (SAEs) were monitored according to Good Clinical Practices. Examples of monitored events include arrhythmias and significant changes in blood pressure.

Concerning teaching, health surveillance needs and cardiovascular risk factors were included, given that due to the established therapy and surgical process, risk factors such as hypertension, dyslipidemia, obesity, and a sedentary lifestyle are very common, with the educational component being fundamental in cardiac rehabilitation programs [20].

**Table 1:** HRN4HTx protocol stages.

Protocol Stages	Interventions
1	Respiratory exercises, calisthenics exercises (assisted passive and active polysegmental mobilizations) * (number of sets/min: 1–2; number of repetitions: 8–12)
2	Breathing exercises, calisthenics exercises (assisted active polysegmental mobilizations) * (number of sets/min: 1–3; number of repetitions: 8–12) Cycle ergometer—5–10 min (1–2 last steps back)
3	Breathing exercises Stretches Cycle ergometer—10–15 min (2–4 lasts back) Resistance exercise for upper limbs *—0.5 kg (number of sets/min: 1–3; number of repetitions: 8–12) Static/dynamic balance training exercise March—5 min Teaching about signs and symptoms of adverse events
4	Breathing exercises Stretches Static/dynamic balance exercises Exercise bike—5 min (without resistance speed—8–12 km/h) Upper limb resistance exercises *—0.5 kg (number of sets: 1–3; number of repetitions: 8–12) March—10 min Teaching about signs and symptoms of adverse events and control of pre-existing or risky cardiovascular risk
5	Breathing exercises, Stretches Bike—10–15 mints Upper limb resistance exercises *—1 kg (number of sets/min: 1–3; number of repetitions: 8–12) Lower limb resistance exercises—resistance bands 5 kg (no. of sets: 1–3; no. of repetitions: 8–12) March—10–20 min Stairs—start with 5 steps, progress up to 2 flights (20 steps) Teaching about signs and symptoms of adverse events and control of pre-existing or risky cardiovascular risk Instruction on post-discharge continuity program

* All upper limb exercises met the joint limits/load limitations defined for people with sternotomy, “Keep move in tube” [21,22].

The following were defined as adverse events resulting from the program, following the recommendations of ACSM [15], the European Society of Cardiology (ESC) [23], and the American Heart Association (AHA) for the practice of exercise or physical activity: arrhythmias; lipothymias; dizziness; signs or symptoms of exercise intolerance, including angina or chest pain, marked dyspnea (modified Borg greater than 5), and intense sweating; diastolic blood pressure (DBP) ≥ 110 mm Hg; and a decrease of >10 mm Hg in systolic blood pressure (SBP) during the program, with increased workload as considered parameters [15,24].

All patients started at level 1, and their progression depended on the assessment of health professionals about hemodynamic behavior, their RPE during rehabilitation sessions, and demonstrated knowledge about safety criteria, being recommended whenever the level started at the lower limit described.

Throughout the evolution of levels, educational measures were introduced targeting warning signs and symptoms and cardiovascular risk factors (previously higher prevalence, sedentary lifestyle).

### 2.3. Outcomes

The results defined to evaluate safety were the number and type of adverse events [15,24] that occurred during the intervention sessions. Concerning functional capacity, the difference in pre- and post-transplant self-care capacity was used, using the Barthel Index and the results of the 6 min walk test [25]. To evaluate its effects, the level of knowledge demonstrated was measured by measuring the gains or lack of knowledge at the date of discharge [26].

### 2.4. Statistical Methods

The non-parametric Mann–Whitney–Wilcoxon tests were used considering the sample value and Spearman correlations, with the significance probability value being defined at *p* < 0.01.

## 3. Results

During this period, 34 people were transplanted, with 19 transplanted people completing HRN4HTx (Table 1); the majority (n-14) were transferred from other hospital services in different regions, already with functional limitations that impacted their independence in self-care at the time of admission (Table 2). It was determined by the research team that access to the best health care should not be conditioned, and therefore, all transplant patients during this period had access to the intervention program.

Of the 15 who were not integrated into the program, 9 died in the immediate postoperative period, 2 suffered a stroke with changes in consciousness, and 3 had 2R rejection.

During integration into the program, two patients suffered postoperative complications; however, they were able to continue the program, with an increase in the number of days of hospitalization.

The program started after extubation, with an average start of approximately 19 (±6.89) hours after transplantation.

There were no adverse events during the sessions, with the average values of the subjective notion of effort being 3.9 (low intensity), with average diastolic pressure values of 76.84 mmHg below the risk value, and with an increasing tendency to increase the systolic blood pressure between assessments (Table 3).

Regarding self-care capacity, patients admitted from other hospital units have greater levels of self-care commitment than those who come from home, with an average difference in Barthel Index scores of 32.21. There was an improvement in all patients, with this being more significant in patients admitted to the hospital, requiring an average of 2 more days of hospitalization to obtain similar levels of self-care capacity.

The average number of days of hospitalization was 15 days, and patients with a greater number of days of hospitalization had similar results in self-care capacity compared to those who had a lower number of days, even though there were two post-transplant complications.

Patients with a higher Barthel Index score on arrival showed better results in the 6 min walk test.

At the time of discharge, all patients and their caregivers had demonstrated knowledge of the warning signs and symptoms and the cardiovascular risk factors to be prevented, regardless of their original knowledge upon admission (Table 4).

## 4. Discussion

The number of heart transplants has stabilized at approximately 5500 procedures worldwide, so the case series has remained similar in recent years [27]. In HRN4HTx, the sample is only 19 people with heart transplants; however, when analyzing studies of structured phase 1 cardiac rehabilitation programs in heart transplantation, samples seen are similar to the study by Kawauchi and collaborators [28] in which 22 transplant recipients were included, divided into two groups of different rehabilitation programs. Some studies refer to inpatient programs in rehabilitation units several days/weeks after hospitalization in a heart transplant unit, with 12 and 17 heart transplant recipients [29,30], or studies with multiple solid organ transplant populations, with 10 heart transplants included [31,32].

Postoperatively, the general objective is to discharge the patient as early as possible. The average length of stay in this study is 15 days, which is lower than some of the published studies, which have an average of between 20 and 35 days [28,33,34,35], which seems to corroborate the importance of the rehabilitation program.

However, this early discharge is not always possible for patients with significant premorbid disability or postoperative complications. Similar to the study by Bowman and Faux [36], the deficit in the ability to carry out activities of daily living was significant pre-transplant; in our sample, the average Barthel Index is 45, which is in line with the findings in which the average Barthel Index is 52.895. These findings demonstrate the importance of early and targeted rehabilitation interventions.

Despite recommendations from international societies, the definition of guidelines with detailed exercise prescriptions for phase 1 heart transplantation is poorly defined or even omitted [14,32]. In the unit where the study took place, there was no program, and only patients who had postoperative complications, such as serious neurological events or myopathy associated with intensive care, were the target of rehabilitation intervention.

The construction of the HRN4HTx stages allowed the application of part of the conceptual recommendations, which, analyzing the particularities of transplant patients’ responses to exercise, demonstrate that it is also applicable using the educational and physical exercise component during the immediate postoperative period (during hospitalization), as recommended by Squires and Bonikowske [20] and Rydberg et al. [16], in their literature reviews. This study demonstrated that this rehabilitation protocol can be safe to implement in the postoperative period of heart transplantation in a transplant unit. There were no adverse events during the rehabilitation interventions.

The educational component is fundamental in rehabilitation programs, especially the elements regarding alarm signs and cardiovascular risk factors, as listed by Kourek et al. [1]. One aspect is the result at the date of discharge of knowledge demonstrated by the sample regarding the domains included in the program. Family involvement in these educational programs improves the response to the rehabilitation program and its outcomes [25].

We have already analyzed that this study presents shorter hospital stays than several studies, but the functional results at the date of discharge must be further explored; as intra-hospital rehabilitation programs are not guaranteed, these patients are unable to demonstrate sufficient improvement in their functional deficits for a return to the home directly from the acute care unit [29]. In this study, a positive evolution in self-care capacity can be seen, with the average Barthel Index of 96 points being close to total independence, which would be a score of 100, given that it is described by Barker et al. [25] as an important indicator of the improvement of functional capacity and the positive evolution of carrying out activities of daily living/self-care. In the study by Joshi and Kevorkian [30], there was no implementation of a rehabilitation program (the transplant population had similar demographic characteristics to those in this study), and with the average Barthel Index score at the date of discharge being 86.5, compared with the results of HRN4HTx, with a Barthel Index of 96, the importance of rehabilitation intervention can be observed.

Concerning functional capacity measured by the 6 min walk test, it is clear that these patients in the immediate postoperative period still present a deficit in capacity when compared to the healthy population, which is in line with what was described by Doutreleau et al. [37]. The study population presented around 54% of the predictive 6 min walk test value, and the study by Kawauchi et al. [28] presents a value of 70%; however, the average number of days of hospitalization is approximately double (32 days) that of HRN4HTx in an intervention program sample of 11 transplant recipients. These data reinforce, in some way, the need for phase 1 cardiac rehabilitation programs in transplant patients.

## 5. Strengths and Limitations of the Study

To date, the research team is not aware of an intervention program with the particularities described in this study, making it internationally innovative and potentially initiating a new area of research in cardiac rehabilitation for hospitalizations in the immediate postoperative period of heart transplantation. This study opens new opportunities to enhance care for these patients using structured programs with the necessary flexibility for individualization as early as possible. It suggests that these programs should be considered pre-transplantation to enhance postoperative capacity gains.

The limitations of this study include the absence of a control group, which limits the ability to attribute the observed outcomes solely to the rehabilitation protocol. Future studies should include a control group receiving standard care for a more robust comparison. Additionally, the study was conducted in a single transplant center, which may limit the generalizability of the findings to other settings with different resources, protocols, and patient populations. Multi-center studies would help to validate the results across various contexts. These limitations led to a retrospective registration in clinical trials, for ethical and scientific transparency reasons, although the number of participants could not be changed since recruitment was only carried out specifically in that hospital center.

The absence of pre-transplant assessments restricts the ability to compare variables pre- and post-transplant, which could provide a more comprehensive understanding of the rehabilitation program’s impact. The relatively small sample size of 19 patients may not be representative of the broader population of heart transplant recipients, affecting the generalizability of the results. Larger sample sizes in future studies would increase the validity of the findings. Differences in pre-transplant care among patients could have influenced the outcomes. Standardizing pre-transplant care or stratifying patients based on their pre-transplant status could provide more accurate assessments of the rehabilitation protocol’s efficacy.

This study focuses on the immediate postoperative period without long-term follow-up data, limiting the understanding of the sustainability of the rehabilitation benefits. Future research should include long-term follow-up to assess the durability of functional improvements and the incidence of late complications. Furthermore, this study does not deeply evaluate psychological and social factors, which can significantly influence rehabilitation outcomes. Future studies should incorporate detailed assessments of these factors to provide a more holistic view of patient recovery. While some patients experienced postoperative complications, this study does not provide a detailed analysis of how these complications affected rehabilitation outcomes. Future research should explore the impact of different types of postoperative complications on rehabilitation efficacy.

By addressing these limitations, future research can build on the findings of this study, providing more comprehensive and generalizable insights into the effectiveness of phase 1 cardiac rehabilitation programs for heart transplant recipients.

## 6. Conclusions

RN4HTx is a feasible cardiac rehabilitation program without adverse events in the immediate postoperative period of heart transplantation, and appears to have a positive impact on functional recovery and therapeutic self-care capacity, increasing the safety of returning home.

## 7. Nursing Implications

Rehabilitation nursing plays a fundamental role in the post-heart-transplant inpatient phase, and integration into different transplant teams should be encouraged. The results of the RN4HTx cardiac rehabilitation program highlight the role of the rehabilitation nurse in improving functional capacity and independence in post-transplant self-care, areas of fundamental nursing intervention.

The structuring of the phase 1 cardiac rehabilitation program is emerging and leads to functional improvements in people with heart transplants; it should begin pre-surgery.

This study demonstrates a path for other investigations into the impact of rehabilitation nursing interventions, consolidating knowledge and supporting clinical practice.

## Figures and Tables

**Table 2:** Comparison of baseline characteristics of patients.

Variables	Inpatient Group (n-19; Age—50, 21 Years Min-18–Max-68 Male 68.42% (n-13) Female 31.58% (n-6)		*p*-Value
Source of admission	**From other wards** 73.68% (n-14)	**From home** 26.32% (n-5)	
Age (years)	51.6 (±9.499) (min—33–max—68)	46.4 (±16.89) (min—18–max—66)	0.643
Gender n (%)			0.520
Male	71.43% (n-10)	60% (n-3)	
Female	28.57% (n-4)	40% (n-2)	
Number of days after heart transplant	15.57 (±5.551)	13.4 (±2.576)	0.485
Barthel on admission	36.79	69	0.027
Postoperative Complications			
Delirium/Encephalopathy	1	0	
Stroke	1		
HRN4HTx	100%	100%	
Adverse events related to HRN4HTx	0	0	

**Table 3:** Safety parameters/adverse events (530 sessions performed).

Adverse Events	Number of Events
Arrhythmias	0
Lipothymias/dizziness	0
Signs/symptoms of exercise intolerance: - Angina/precordialgia - Subjective notion of effort—modified Borg Intense sweating	0 0 0 (Average values of 3.9) 0
Diastolic blood pressure (DBP) ≥ 110 mm Hg	0 (Average DBP values of 76.84 mmHg)
Decrease of >10 mm Hg systolic blood pressure (SBP)	0 (Average SBP change values of −5.74 mmHg)

**Table 4:** Functional capacity and self-care data.

Origin (n-19)	Hospital Department	Home	*p*-Value	Inpatient Group
Barthel on admission	36.79 (±21.011)	69 (±18.276)	0.027	52.895
Barthel on discharge	95 (±4.629)	97 (±4)	0.420	96
Walking test—6 min (meters)	271.07	349	0.194	310.035
Demonstrated Knowledge about:				
- Signs and symptoms of alarm	100%	100%		
- Cardiovascular risk factors	100%	100%		

## Data Availability

Data are contained within the article.

## Public Involvement Statement

Patients were involved in this research as participants. They provided written consent before participating; their experiences and outcomes were essential to the study’s findings. However, there was no direct involvement of patients, consumers, or carers in our research’s design, conduct, reporting, or dissemination plans.

## Guidelines and Standards Statement

This manuscript was drafted against the TREND (Transparent Reporting of Evaluations with Nonrandomized Designs) guidelines for reporting non-randomized intervention studies.
